# HCMV infection downregulates GPX4 and stimulates lipid peroxidation but does not induce ferroptosis

**DOI:** 10.1128/jvi.01851-24

**Published:** 2025-01-07

**Authors:** Madison Martin, Rinki Kumar, Nicholas J. Buchkovich, Christopher C. Norbury

**Affiliations:** 1 Department of Microbiology and Immunology, Penn State College of Medicine 219261 , Hershey, Pennsylvania, USA; Northwestern University Feinberg School of Medicine, Chicago, Illinois, USA

**Keywords:** HCMV, iron, lipid peroxides, ferroptosis

## Abstract

Human cytomegalovirus (HCMV) modulates numerous cellular pathways to facilitate infection, including key components in cellular iron homeostasis. Iron is essential to many cellular processes but, if present in excess, drives cell death through ferroptosis. Ferroptosis is a process that is dependent upon the accumulation of oxidatively damaged phospholipids (lipid peroxides); when these lipid peroxides accumulate in membranes, this culminates in plasma membrane rupture and eventual cell lysis. Here, we demonstrate that HCMV infection downregulates the expression of a key modulator of lipid peroxidation, glutathione peroxidase 4 (GPX4). HCMV infection also markedly increased levels of lipid peroxides within infected cells. Despite the marked downregulation of GPX4 by HCMV, further inhibition of GPX4 impaired virus replication. Interestingly, overexpression of GPX4 did not reduce the production of lipid peroxides within infected cells. In contrast, lipid peroxide levels were reduced by treatment with ferrostatin-1, a ferrous iron-dependent scavenger of alkoxyl radicals, indicating a role for iron in the production of lipid peroxides. HCMV-infected cells became less sensitive to GPX4 inhibition as infection progressed, requiring substantially higher levels of GPX4 inhibitors to induce ferroptosis compared to uninfected cells. This observed difference in sensitivity to ferroptosis upon infection correlated with a large increase in lipid production by infected cells. Therefore, the marked stimulation of lipid peroxidation by HCMV likely proceeds through a pathway that is independent of GPX4 regulation, but the ability of lipid peroxides to stimulate ferroptosis by modulating plasma membrane rupture is likely blunted by the massive increase in lipid production during HCMV infection.

**IMPORTANCE:**

Human cytomegalovirus (HCMV) infection is intimately linked with countless host cell pathways that are modulated in a coordinated fashion to facilitate infection. Here, we describe HCMV-induced regulation of lipid peroxidation, a precursor of the iron-regulated cell death pathway known as ferroptosis, during human cytomegalovirus infection. These studies reveal hitherto unidentified changes in metabolism mediated by HCMV that decrease sensitivity to ferroptosis, despite increases in lipid peroxidation and transient increases in intracellular iron levels in infected cells.

## INTRODUCTION

Human cytomegalovirus (HCMV) is a betaherpesvirus that causes disease in immunocompromised patients and fetal birth defects after congenital infection. There are no approved vaccines or non-toxic treatment options. HCMV infection drastically alters the cellular environment and utilizes host pathways, including the reorganization of membranous organelles and the modulation of host innate immune responses, to facilitate infection. HCMV is also known to modulate stress responses and subvert cell death pathways during replication (1–10). Finally, HCMV also significantly changes cellular metabolism, markedly increasing lipid biogenesis in the late stages of infection (11–13). Investigation of the mechanisms required to modulate host pathways may allow for the identification of new therapeutic targets.

We have previously shown that appropriate regulation of iron is crucial for efficient HCMV infection, with iron levels increasing early in infection and returning to normal late in infection (14). Interestingly, maintenance of the high intracellular iron levels that are produced early during infection into the later phase of infection reduced virus replication (14), implying that sustained high iron levels were toxic to HCMV. Ferroptosis is an iron-regulated programed cell death pathway in which accumulation of excess iron drives free radical formation, causing oxidation of lipids along with other morphological, transcriptional, and translational changes, which together lead to cell death (15, 16). It is possible that prolonging elevated iron levels into later stages of HCMV infection could induce ferroptosis-mediated death of HCMV-infected cells that leads to reduced viral titers. While HCMV is known to encode gene products to counteract both apoptosis and necroptosis (7, 17–19), the regulation of ferroptosis during HCMV infection has not been previously described. Therefore, we sought to examine mechanisms that regulate lipid peroxidation and the potential induction of ferroptosis during HCMV infection.

HCMV infection causes an increase in lipid peroxidation, a key driver and indicator of ferroptosis (14). When lipid peroxidation accumulates in biological membranes, it can lead to decreased membrane flexibility and thickness and eventually cause membrane rupture (20–23). Two proteins involved in protection against ferroptosis were both downregulated during a wild-type HCMV infection (14). Ferritin heavy chain, which normally prevents accumulation of excess iron (a driver of ferroptosis), is downregulated by HCMV infection. HCMV also downregulates glutathione peroxidase 4 (GPX4), a glutathione-dependent hydroperoxidase that reduces lipid peroxides to form non-toxic lipid alcohols and, therefore, prevents accumulation of the lipid peroxides that drive ferroptosis (24). The increase in lipid peroxidation and downregulation of these protective mechanisms during infection suggests that HCMV-infected cells may be sensitive to ferroptosis-mediated cell death, which would present an intriguing new target for therapeutic development.

To gain a better understanding of the regulation of ferroptosis during HCMV infection, we studied the modulation of lipid peroxidation during infection by investigating two enzymes responsible for controlling lipid peroxidation: GPX4 and ferroptosis suppressor protein 1 (FSP1, also known as apoptosis-inducing mitochondria-associated 2). FSP1 was originally identified as an enzyme that controls ferroptosis in cells lacking GPX4 and acts by reducing ubiquinone to ubiquinol, which in turn reduces oxidized lipids (25, 26). While both GPX4 and FSP1 have been identified as regulators of lipid peroxidation and ferroptosis, depletion of GPX4 alone induces ferroptosis, whereas FSP1 is only required in the absence of GPX4 expression (24–26). Here, we examine the expression and regulation of these proteins, explore their role in productive infection, and investigate their function in the regulation of both lipid peroxidation and the induction of ferroptosis during HCMV infection. We found that HCMV induces lipid peroxidation in an iron-dependent manner independent of both GPX4 and FSP1, and these increased levels of lipid peroxides do not induce ferroptosis in HCMV-infected cells.

## RESULTS

### Lipid peroxidation and its regulators are differentially modulated during HCMV infection

**Fig 1:**
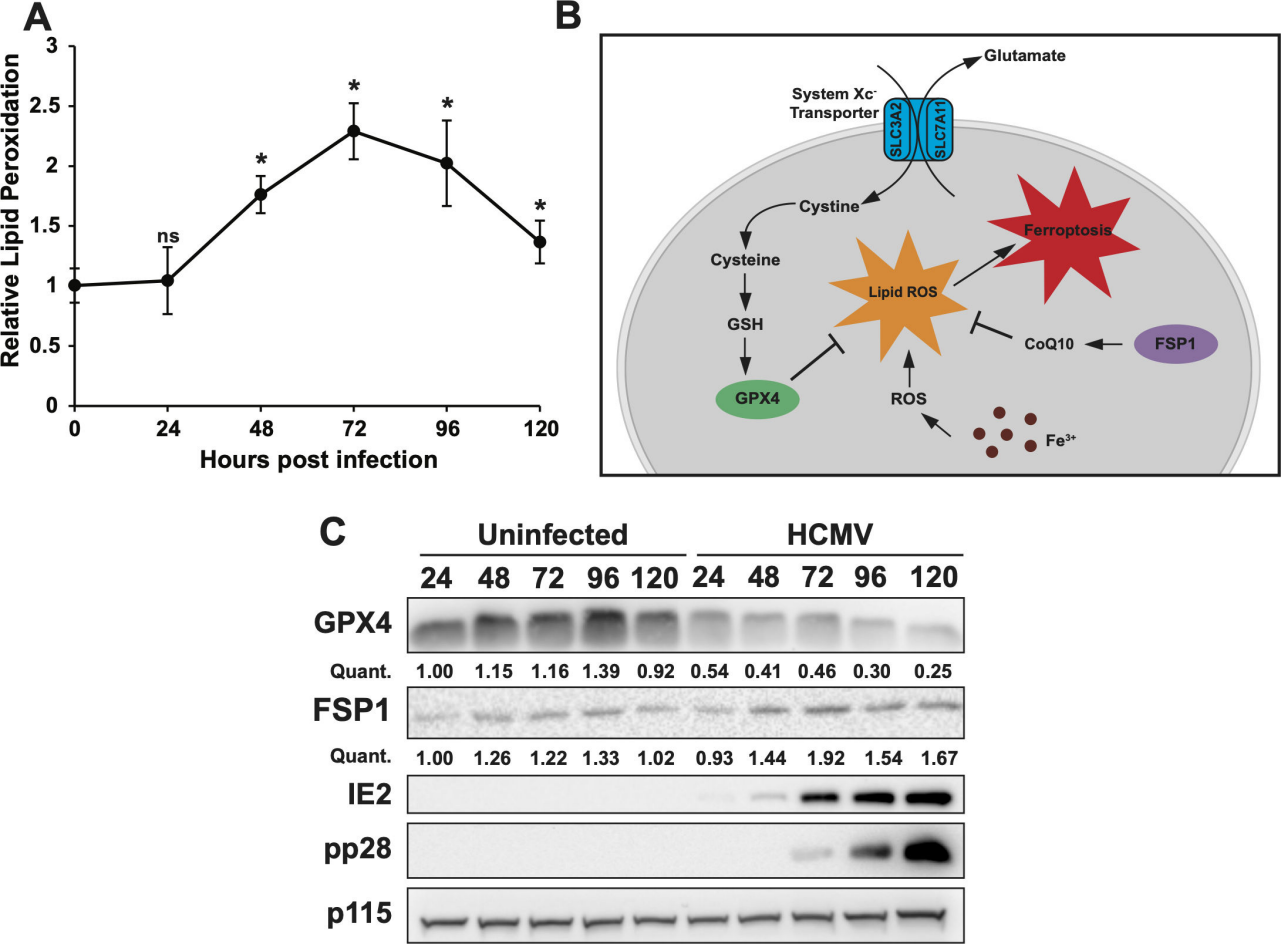
HCMV differentially regulates GPX4 and FSP1 during infection. (**A**) Lipid peroxidation measured with C11 BODIPY FITC fluorescence shift in MRC-5 cells either uninfected (0 hpi) or TB40/E-WT (MOI = 3) infected at 24, 48, 72, 96, and 120 hpi. Lipid peroxidation is shown relative to uninfected cells. Results are from two individual experiments, *n* = 6. (**B**) Model depicting the pathways of GPX4 and FSP1 to reduce lipid peroxidation and regulate ferroptosis. (**C**) Western blot analysis of lysates from uninfected MRC5 cells or MRC5 cells infected with TB40/E-WT (as in panel A) and harvested at 24, 48, 72, 96, and 120 hours. Blots were probed for GPX4, FSP1, IE2, pp28, and p115 as a loading control. The values under the GPX4 and FSP1 blots denote quantification of each GPX4 or FSP1 band in the blot, relative to the GPX4 or FSP1 band in the uninfected cells at 24 hours, respectively. For the above panels, * indicates *P* < 0.05 and ns = not significant. MOI, multiplicity of infection.

We have previously demonstrated that HCMV infection drives an increase in lipid peroxidation (14). To further explore viral regulation of lipid peroxidation, we examined lipid peroxidation levels over a time course of infection. At 24 hours post-infection (hpi), lipid peroxidation was unchanged, but by 48 hpi, it had increased significantly (Fig. 1A). Lipid peroxidation peaked at over double normal levels at 72 hpi, and although it decreased at later time points, it remained elevated above uninfected levels (Fig. 1A). Lipid peroxidation in uninfected cells was equivalent at 0, 24, and 72 hours. In uninfected cells, lipid peroxidation is regulated by GPX4 and, in the absence of GPX4, by FSP1 (Fig. 1B). As HCMV increased lipid peroxidation, we examined the expression of the regulatory proteins GPX4 and FSP1 over time in uninfected or HCMV-infected cells. Expression of the general vesicular transport protein p115 (USO1) remains unaltered throughout infection (27) and thus was used as a loading control. We found that GPX4 and FSP1 are differentially regulated during HCMV infection. We previously showed that GPX4 protein expression decreased considerably by 24 hpi and have now expanded on these findings to show that GPX4 levels remain low throughout infection (Fig. 1C). In contrast, GPX4 expression does not change during the same amount of time in uninfected cells (Fig. 1C). Meanwhile, FSP1 expression increased by 24 hpi and remained high throughout the rest of the infection; however, levels remained low over the same amount of time in uninfected cells (Fig. 1C). This raised the possibility that an increase in FSP1 production could potentially counteract the effects of a decrease in GPX4 expression during HCMV infection.

**Fig 2:**
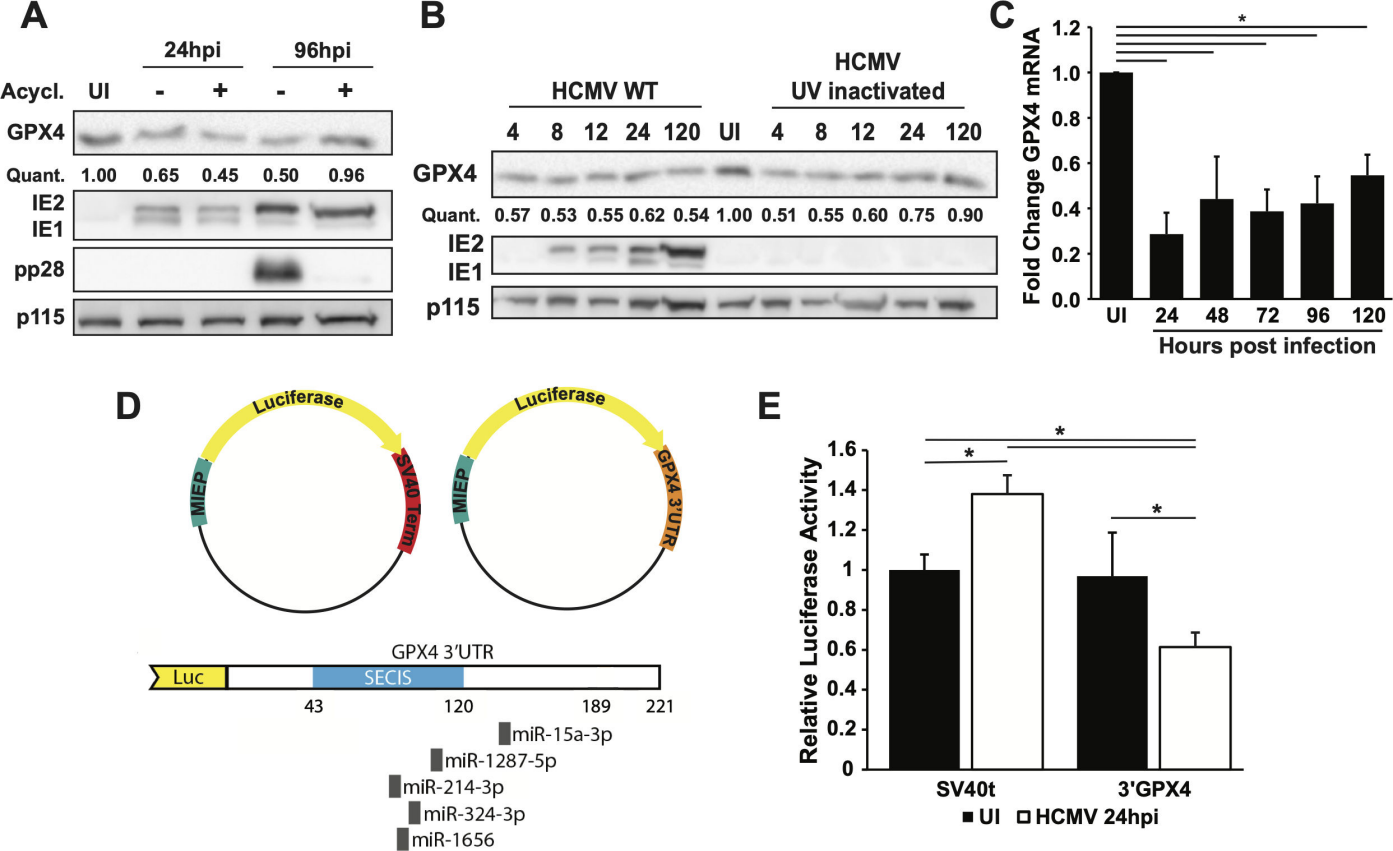
GPX4 regulation occurs in part through the 3′UTR. (**A**) Western blot analysis of lysates from uninfected MRC-5 cells and MRC-5 cells infected with TB40/E-WT virus at MOI = 3, treated with vehicle control (−) or acyclovir (+), and harvested at 24 or 96 hpi. Blots were probed for GPX4, IE1, IE2, pp28, and p115 as a loading control. The values under the GPX4 blot denote quantification of each GPX4 band in the blot, relative to the GPX4 band in the uninfected cells. (**B**) Western blot analysis of lysates from uninfected MRC-5 cells or MRC-5 cells infected with TB40/E-WT virus or UV-inactivated TB40/E-WT virus at MOI = 3 and harvested at 4, 8, 12, 24, and 120 hpi. Blots were probed for GPX4, IE1, IE2, and p115 as a loading control. The values under the GPX4 blot denote quantification of each GPX4 band in the blot, relative to the GPX4 band in the uninfected cells. (**C**) qPCR analysis of GPX4 transcripts isolated from uninfected or TB40/E-WT infected human dermal fibroblasts (MOI = 3) at 24, 48, 72, 96, and 120 hpi. GPX4 transcripts were normalized to GAPDH and shown as fold change relative to the uninfected sample. Results are from four independent experiments. (**D**) Figure portraying the cloning of a control SV40 terminator sequence or GPX4 3′UTR sequence into the luciferase plasmid. The GPX4 3′UTR sequence is shown with notable elements and miRNA-binding sites marked. SECIS denotes the selenocysteine insertion sequence, a component of the GPX4 sequence that is required for its translation. (**E**) Luciferase activity of MRC-5 cells expressing a luciferase control plasmid or a luciferase GPX4 3′UTR plasmid and either uninfected or infected with TB40/E-WT at MOI = 3 and harvested at 24 hpi. Luciferase activity is shown relative to uninfected MRC-5 cells expressing the luciferase control plasmid. Results are from two independent experiments, *n* = 6. For the above panels, * indicates *P* < 0.05 and ns = not significant. qPCR, quantitative PCR.

To investigate the mechanism of GPX4 downregulation during HCMV infection, infected cells were treated with acyclovir (100 µg/mL) at 3 hpi to block viral genome replication and late gene expression. Despite acyclovir treatment, GPX4 was still downregulated at 24 hpi (Fig. 2A). GPX4 expression returned to close to pre-infection levels at 96 hpi with acyclovir treatment, likely because inhibition of viral replication allowed GPX4 expression to return to normal (Fig. 2A). Consistent with this, GPX4 downregulation also occurred following exposure of cells to UV-inactivated HCMV, indicating that viral gene expression is not required for GPX4 downregulation (Fig. 2B). Additionally, GPX4 downregulation occurred as early as 4 hpi in both wild-type and UV-inactivated HCMV infection (Fig. 2B). To investigate the potential for viral transcriptional regulation of GPX4, we examined mRNA levels for GPX4 and found that GPX4 expression is decreased throughout infection by ~50% (Fig. 2C). GPX4 expression is regulated via microRNA (miRNA) binding to the 3′UTR in different cancer models, including lung, colorectal, prostate, and liver cancer (28–31). Active binding sites of multiple miRNAs have been mapped to the 3′UTR of GPX4 (Fig. 2D). The rapid downregulation of GPX4, which exhibits a half-life of ~24 hours in uninfected cells, and the reduced mRNA level may indicate multiple mechanisms for GPX4 downregulation, including at the mRNA level as has been shown in the cancers mentioned above. To test mRNA regulation, we inserted the GPX4 3′UTR into a luciferase vector following the luciferase gene, and a control plasmid containing the SV40 terminator sequence was also generated (Fig. 2D). Following transduction of these luciferase plasmids into fibroblasts, cells with either plasmid expressed similar levels of luciferase. However, when infected, cells expressing the GPX4 3′UTR demonstrated reduced luciferase activity compared to control cells, suggesting a role for 3′UTR regulation of GPX4 expression (Fig. 2E). These results indicate the possibility of miRNA regulation of GPX4 during infection. However, further investigation is required to determine the exact mechanisms of GPX4 downregulation during HCMV infection.

### HCMV alters cellular pathways that control ferroptosis in infected cells

**Fig 3:**
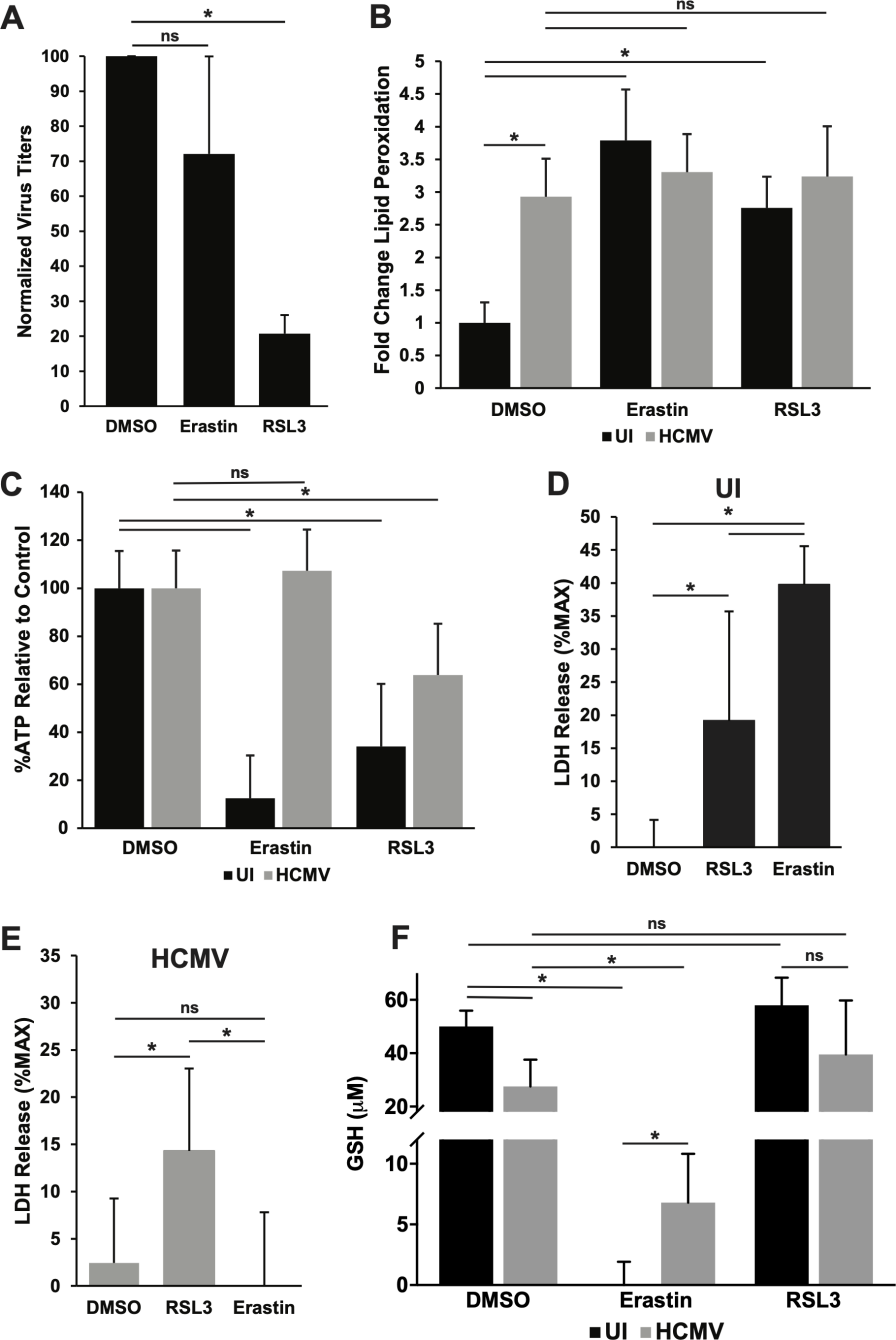
Reduced levels of GPX4 impair infection and induce ferroptosis. (**A**) Infectious titers at 120 hpi from TB40/E-WT (MOI = 3) infected MRC-5 cells treated with vehicle control (DMSO), erastin (5 µM), or RSL3 (1 µM) at 72 hpi. Results are from three independent experiments, *n* = 4. (**B**) Lipid peroxidation was measured with C11 BODIPY FITC fluorescence shift in MRC-5 cells either uninfected or infected (as in panel A), treated with DMSO, erastin, or RSL3, and harvested at 96 hpi. Infected cells were treated for 24 hours before harvest, while uninfected cells were treated for 14 hours (DMSO and erastin) or 4 hours (RSL3). Lipid peroxidation is shown relative to uninfected, DMSO-treated cells. Results are from three independent experiments, *n* = 6. (**C**) ATP levels at 96 hpi in uninfected MRC-5 cells treated with DMSO, erastin, or RSL3 as described in (**A**), or MRC-5 cells infected with TB40/E-WT and treated as described in (**A**). Data are shown as %ATP relative to DMSO-treated cells in the uninfected or HCMV-infected group. Results are from two independent experiments, *n* = 6. (**D**) LDH release in uninfected MRC-5 cells treated with DMSO, erastin, or RSL3 for 24 hours. LDH release is shown as the percent of maximum LDH in uninfected cells. Results are from three independent experiments, *n* = 9. (**E**) LDH release in MRC-5 cells infected with HCMV (as in panel A) and treated with DMSO, erastin, or RSL3 at 72 hpi and measured at 96 hpi. LDH release is shown as the percent of total LDH in infected cells. Results are from three independent experiments, *n* = 9. (**F**) GSH levels measured in uninfected or TB40/E-WT (MOI = 3) infected MRC-5 cells at 96 hpi following treatment with DMSO, erastin, or RSL3. Infected cells were treated for 24 hours before harvest, while uninfected cells were treated for 14 hours (DMSO and erastin) or 4 hours (RSL3). Results are from three independent experiments, *n* = 8. For the above panels, * indicates *P* < 0.05 and ns = not significant.

The concurrent downregulation of GPX4 and increase in lipid peroxidation during infection suggests that HCMV-infected cells may be sensitized to ferroptosis. To examine the role of GPX4 in infection, we titrated HCMV-infected cells with two compounds that target the GPX4 pathway at different points. Erastin, one of the first discovered ferroptosis-inducing compounds, inhibits system Xc^−^, a transporter that imports cystine into the cell. In the absence of cystine, glutathione (GSH) synthesis is impaired, thus reducing substrate levels for GPX4 and impairing GPX4 activity (15, 16) (Fig. 1B). RSL3, which also induces ferroptosis, acts directly upon GPX4 to inhibit its function (24, 32). Treatment with RSL3 reduced virus titers in a high MOI infection by greater than sixfold relative to dimethyl sulfoxide (DMSO) vehicle treatment in each experiment, indicating a role for GPX4 in infection (Fig. 3A). However, treatment with erastin did not decrease viral titers (Fig. 3A), suggesting that HCMV may modulate some factors in the pathway between system Xc^−^ and GPX4, the respective targets of erastin and RSL3. Although RSL3 has been shown to have off-target effects on another selenocysteine protein, thioredoxin reductase (TXNRD) (33), we found that its effect on virus replication was independent of its effect on TXNRD activity (Fig. S1). While both RSL3 and a specific TXNRD inhibitor, Tri-1 (34), inhibited TXNRD activity, only RSL3 had an effect on HCMV replication (Fig. S1).

As RSL3 and erastin both induce ferroptosis, and we have previously shown that the induction of ferroptosis reduces virus replication, we examined whether RSL3 and erastin-treated cells exhibited any evidence of ferroptosis. Ferroptotic cells undergo cell death due to an accumulation of lipid peroxides. Increased lipid peroxidation is both a hallmark and a driver of ferroptosis, while decreased ATP levels and membrane integrity are also common indicators of cells undergoing ferroptosis (35–37). However, it should be noted that neither increased lipid peroxidation nor decreased ATP levels or membrane integrity are specific to ferroptotic cell death (35–37). We measured the accumulation of lipid peroxides with C11 BODIPY, a lipophilic dye that shifts fluorescence of lipids from red fluorescence to green upon the formation of lipid peroxides after oxidation of those lipids (36). As previously reported, infected cells exhibited increased lipid peroxidation compared to uninfected cells (Fig. 3B). As expected, treatment with the ferroptosis-inducing compound erastin increased lipid peroxidation in uninfected cells (Fig. 3B). However, erastin treatment of HCMV-infected cells did not lead to a further accumulation of lipid peroxides (Fig. 3B). Similarly, RSL3 treatment increased lipid peroxidation in uninfected cells but did not further increase lipid peroxidation in HCMV-infected cells (Fig. 3B). Thus, neither ferroptosis-inducing compound increased lipid peroxidation in HCMV-infected cells.

To examine the effects of HCMV, erastin, and RSL3 upon cell viability, we measured ATP levels using a Cell Titer-Glo cell assay. As expected, erastin treatment drastically reduced ATP levels in uninfected cells but had no effect on ATP levels in HCMV-infected cells (Fig. 3C). In contrast to erastin, RSL3 treatment caused a reduction in ATP levels in both uninfected and infected cells (Fig. 3C). Therefore, similar to the data shown in Fig. 3A, in which erastin and RSL3 have distinct effects upon HCMV replication, the differential ability of these compounds to modulate cell viability suggests that HCMV modulates the pathway between system Xc^−^ and GPX4, the targets of erastin and RSL3, respectively.

Membrane integrity was evaluated by measuring LDH release using a CyQUANT assay, in which increased release of LDH into the media indicates a loss of membrane integrity. Erastin treatment alone triggered a significant increase in LDH release compared to vehicle-treated cells. However, in infected cells, erastin treatment did not alter LDH release compared to vehicle-treated, infected cells (Fig. 3D and E). In contrast, and similar to its effect upon ATP levels, RSL3 increased LDH release compared to vehicle treatment in both uninfected and infected cells (Fig. 3D and E). Taken together, these data indicate that HCMV infection alone increased some of the hallmarks of ferroptosis. Further decreasing GPX4 activity during infection via RSL3 treatment decreased both ATP levels and membrane integrity but did not increase the hallmark driver of ferroptosis, lipid peroxidation (Fig. 3B through E). This indicates that residual activity of the remaining GPX4 within infected cells during HCMV infection can still likely modulate cell viability, although a direct linkage to ferroptosis has not been established. Interestingly, erastin treatment did not mimic the effects of RSL3 upon infected cells (Fig. 3B through E). Therefore, HCMV likely modulates components of the host cellular pathway between system Xc^−^, which is inhibited by erastin, and GPX4, which is inhibited by RSL3.

To investigate whether HCMV infection modulates the activity of the metabolic pathway between system Xc^−^ and GPX4, we sought to explore the effects of erastin and RSL3 on intracellular levels of GSH, the substrate of GPX4 used to reduce lipid peroxides. Erastin is a small molecule that acts by inhibiting the function of the system Xc^−^ transporter, which imports cystine into the cells. Erastin treatment inhibits cystine import, leading to cysteine starvation, glutathione depletion, and subsequent inhibition of GPX4, which in turn causes ferroptotic cell death (15, 32, 38). RSL3 treatment directly inhibits GPX4 and thus should not measurably affect glutathione levels. It has previously been reported that reduced thiols, which include GSH, are increased following HCMV infection (39). However, when we directly measured GSH levels, rather than reduced thiols, in uninfected or HCMV-infected cells, we observed a ~50% decrease in intracellular GSH concentration in infected cells (Fig. 3F). We then examined GSH levels in infected or uninfected cells treated with RSL3 and erastin. As expected, we found that RSL3 caused a small increase in GSH levels that did not achieve statistical significance (Fig. 3F). In contrast, erastin treatment of uninfected fibroblasts reduced GSH levels to background levels. Surprisingly, however, erastin treatment of infected cells did not completely deplete GSH levels, which were reproducibly detectable and significantly increased compared to uninfected cells treated with erastin (Fig. 3F). Therefore, it appears that HCMV-infected cells may downregulate conventional erastin-sensitive GSH production, while simultaneously upregulating an alternative erastin-insensitive mechanism, thus potentially altering the sensitivity of cells to ferroptotic cell death.

### GPX4 knockdown inhibits viral infection and increases ferroptotic markers

**Fig 4:**
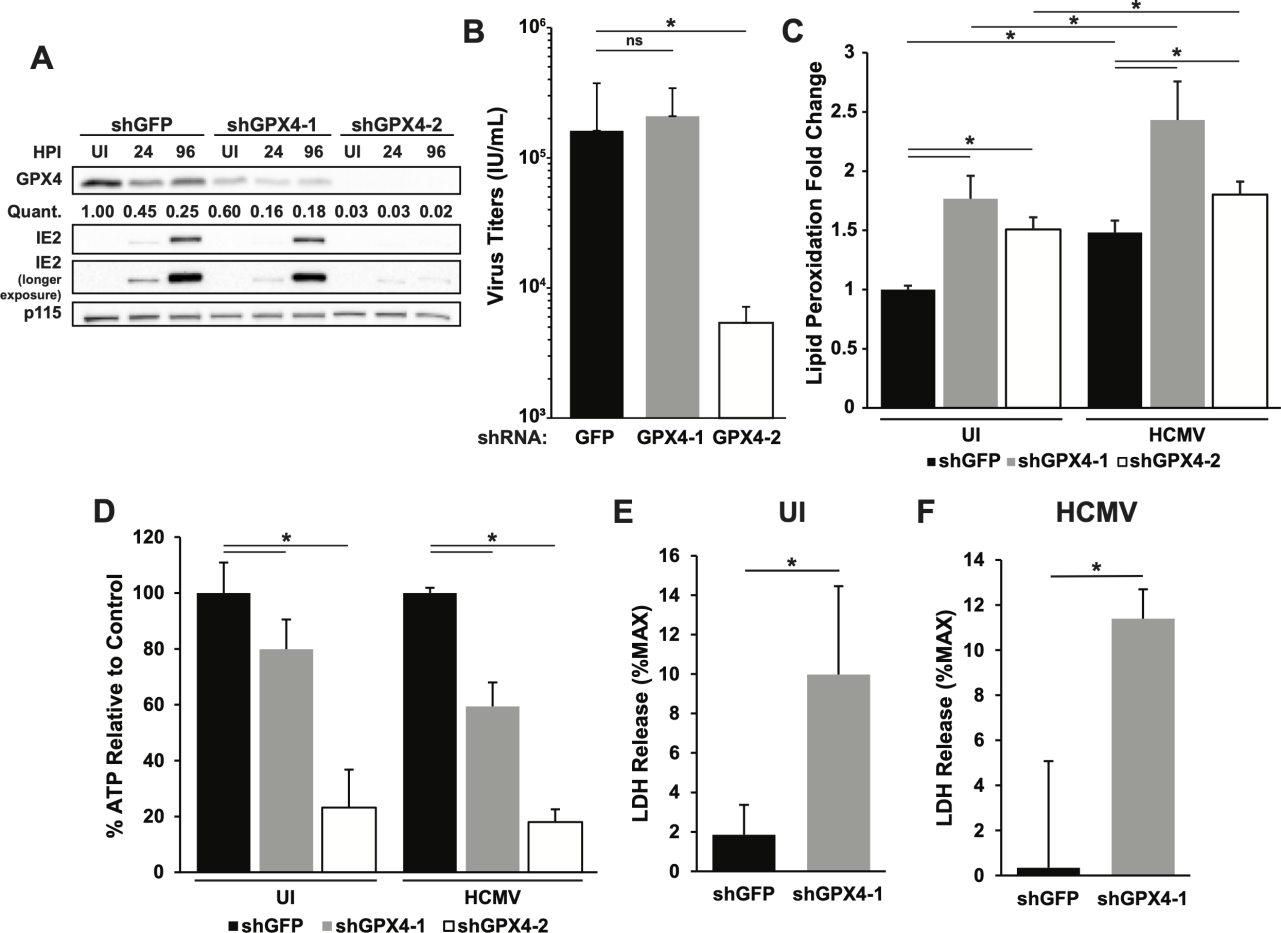
GPX4 knockdown inhibits viral infection and increases ferroptosis markers. (**A**) Western blot analysis of MRC-5 cells expressing control shRNA (shGFP) or GPX4 shRNA (shGPX4-1 and shGPX4-2). Cells were either uninfected or infected with TB40/E-WT at MOI = 3 and harvested at 24 or 96 hpi. Blots were probed for GPX4, IE2, and p115 as a loading control. The values under the GPX4 blot denote quantification of each GPX4 band in the blot, relative to the GPX4 band in shGFP uninfected cells. (**B**) Infectious titers at 120 hpi from HCMV-infected (as described in panel A) MRC-5 cells expressing shRNA against green fluorescent protein (GFP) or GPX4. Results are from five independent experiments, *n* = 15. (**C**) Lipid peroxidation measured by C11 BODIPY FITC fluorescence shift in MRC-5 cells expressing shGFP or either shGPX4 clone either uninfected or infected with TB40/E-WT (MOI = 3) at 96 hpi. Data are shown relative to control (shGFP) uninfected cells. Results are from two independent experiments, *n* = 6. (**D**) ATP levels in MRC-5 cells expressing control shGFP or shGPX4, either uninfected or HCMV-infected (as described in panel A) and measured at 96 hpi. ATP levels are shown relative to respective uninfected or infected shGFP-expressing cells. Results are from two independent experiments *n* = 8. (**E**) LDH release in uninfected MRC-5 cells expressing shRNA against GFP or GPX4. LDH release is shown as the percent of maximum LDH in uninfected control cells. Results are from two independent experiments, *n* = 6. (**F**) LDH release in MRC-5 cells expressing shRNA against GFP or GPX4 and infected with HCMV (as in panel A) and measured at 96 hpi. LDH release is shown as the percent of maximum LDH in infected cells. Results are from two independent experiments, *n* = 6. For the above panels, * indicates *P* < 0.05 and ns = not significant.

In the above experiments, we used chemical inhibitors to probe the production of lipid peroxides and the induction of ferroptosis in HCMV-infected cells. As chemical inhibitors can often have off-target side effects, we generated cells in which expression of GPX4 was targeted with short hairpin RNA (shRNA) in order to increase the rigor of our results. Two shRNA clones that were introduced into MRC-5 cells via lentiviral transduction produced different levels of knockdown (Fig. 4A). Following transduction with sh-GPX4 clone 1, GPX4 knockdown was mild, allowing infection and virus production to proceed similarly to control shGFP-expressing cells (Fig. 4A and B). Transduction with sh-GPX4 clone 2 produced a dramatic knockdown of GPX4. Clone 2 transduced cells produced very little immediate early 2 (IE-2) protein after HCMV infection and exhibited almost a two-log reduction in viral titers (Fig. 4A and B). Because clone 2 had such a significant knockdown, these cells divided slowly and were only viable for a limited number of passages, making them difficult to use for all assays. It is unsurprising that such a dramatic knockdown of GPX4 as seen with clone 2 would have such an effect on cell division because of the integral role of GPX4 in maintaining redox homeostasis. While this could affect the interpretation of the results seen with the GPX4 clone 2 knockdown cells, our data are corroborated by our results with RSL3-mediated inhibition of GPX4. Therefore, we used clone 1, as well as clone 2 where possible, for all subsequent assays. Compared to uninfected control cells, GPX4 knockdown cells exhibited higher baseline levels of lipid peroxidation (Fig. 4C), reduced basal levels of ATP (Fig. 4D), and increased LDH release (Fig. 4E), indicative of reduced cell viability. Thus, it is likely that at baseline, cells expressing less GPX4 are more sensitive to the induction of ferroptosis. Following HCMV infection, we observed that GPX4 knockdown further increased lipid peroxidation and LDH release but showed a similar decrease in ATP levels to uninfected controls expressing the same shRNA constructs (Fig. 4C, D and F). Overall, these results are similar but not identical to those observed following RSL3 treatment. However, the results demonstrate that during HCMV infection, GPX4 can moderately modulate lipid peroxidation and potentially affect the induction of ferroptosis.

### FSP1 knockdown does not induce ferroptosis in HCMV-infected cells

**Fig 5:**
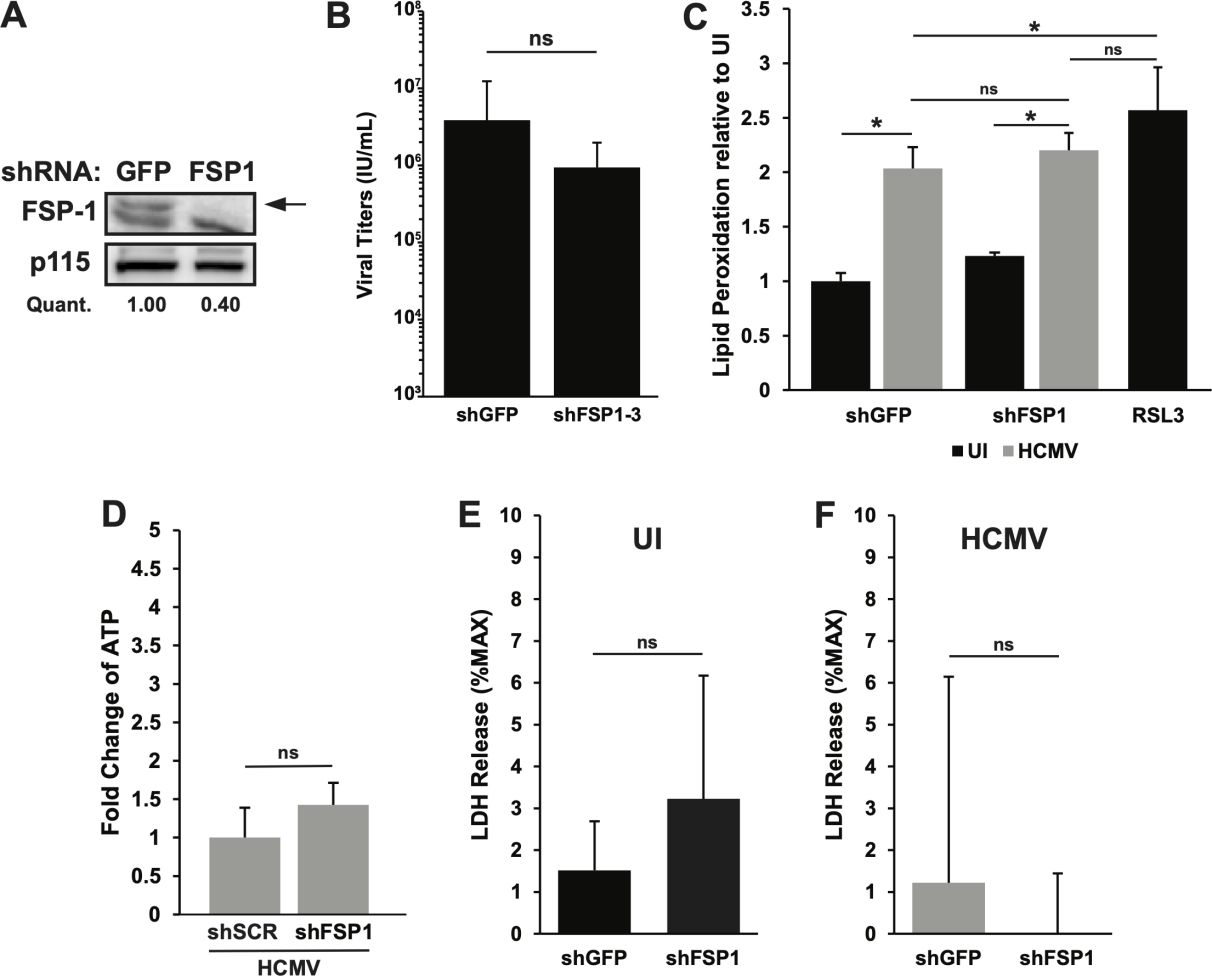
FSP1 knockdown does not impair infection or induce ferroptosis. (**A**) Western blot analysis of lysates from uninfected MRC-5 cells expressing control shRNA (green fluorescent protein [GFP]) or shFSP1 (shRNA against FSP1). Blots were probed for FSP1 and p115 as a loading control. The values under the blot denote quantification of each FSP1 band in the blot, relative to the FSP1 band in shGFP cells. (**B**) Infectious titers at 120 hpi from MRC-5 cells expressing GFP control shRNA or shFSP1 and infected with TB40/E-WT at MOI = 3. Results are from seven independent experiments, *n* = 18. (**C**) Lipid peroxidation was measured by C11 BODIPY FITC fluorescence shift in MRC-5 cells expressing shGFP or shFSP1 and either uninfected or infected as described in panel **B** at 96 hpi. MRC-5 cells treated with RSL3 (1 µM) were used as a positive control. Data are shown relative to uninfected shGFP cells. Results are from two independent experiments, *n* = 6. (**D**) ATP levels in MRC-5 cells infected as described in panel **B** at 96 hpi with cells expressing scrambled shRNA or shFSP1. Data are shown relative to shSCR cells. Results are from two independent experiments, *n* = 9. (**E**) LDH release in uninfected MRC-5 cells expressing shRNA against GFP or FSP. LDH release is shown as the percent of maximum LDH in uninfected control cells. Results are from three independent experiments, *n* = 9. (**F**) LDH release in MRC-5 cells expressing shRNA against GFP or GPX4 and infected with HCMV (as in panel A) and measured at 96 hpi. LDH release is shown as the percent of maximum LDH in infected cells. For the above panels, * indicates *P* < 0.05 and ns = not significant. Results are from three independent experiments, *n* = 9. For the above panels, * indicates *P* < 0.05 and ns = not significant.

It has been reported that, in the absence of GPX4, FSP1 can modulate lipid peroxidation and the induction of ferroptosis. Therefore, we investigated whether FSP1 can play this role in HCMV-infected cells in which GPX4 is partially downregulated (25, 26). To this end, we performed an shRNA knockdown of FSP1 by transducing MRC-5 cells with a lentiviral vector expressing an FSP1-targeting shRNA. Since infected cells have reduced GPX4 levels, we hypothesized that a reduction in FSP1 levels would induce ferroptosis in infected cells, thus inhibiting HCMV infection. We verified that one of the sh-FSP1 clones that we used successfully knocked down FSP1 (Fig. 5A). However, compared to cells transduced with a control shGFP lentiviral vector, the cells in which we observed a marked knockdown of FSP1 did not exhibit a significant reduction in HCMV titers (Fig. 5B). Additionally, we sought to gauge the ability of FSP1 knockdown to induce the hallmarks of ferroptosis in HCMV-infected cells using the assays outlined above. As before, HCMV infection increased lipid peroxidation in MRC-5 cells, but knockdown of FSP1 caused no further increase in lipid peroxidation in the infected cells (Fig. 5C). RSL3 treatment was used as a positive control for lipid peroxidation, and its inclusion in the assay demonstrates that lipid peroxidation had not been saturated in infected cells (Fig. 5C). Consistent with a failure to induce other hallmarks of ferroptosis, FSP1 knockdown did not decrease ATP levels from those found in infected cells (Fig. 5D) or further compromise membrane integrity in uninfected or infected cells (Fig. 5E–F). These results indicate that contrary to our hypothesis, FSP1 knockdown did not induce ferroptosis in infected cells, suggesting FSP1 does not play a role in regulating lipid peroxidation during HCMV infection.

### Lipid peroxidation is differentially regulated during HCMV infection

**Fig 6:**
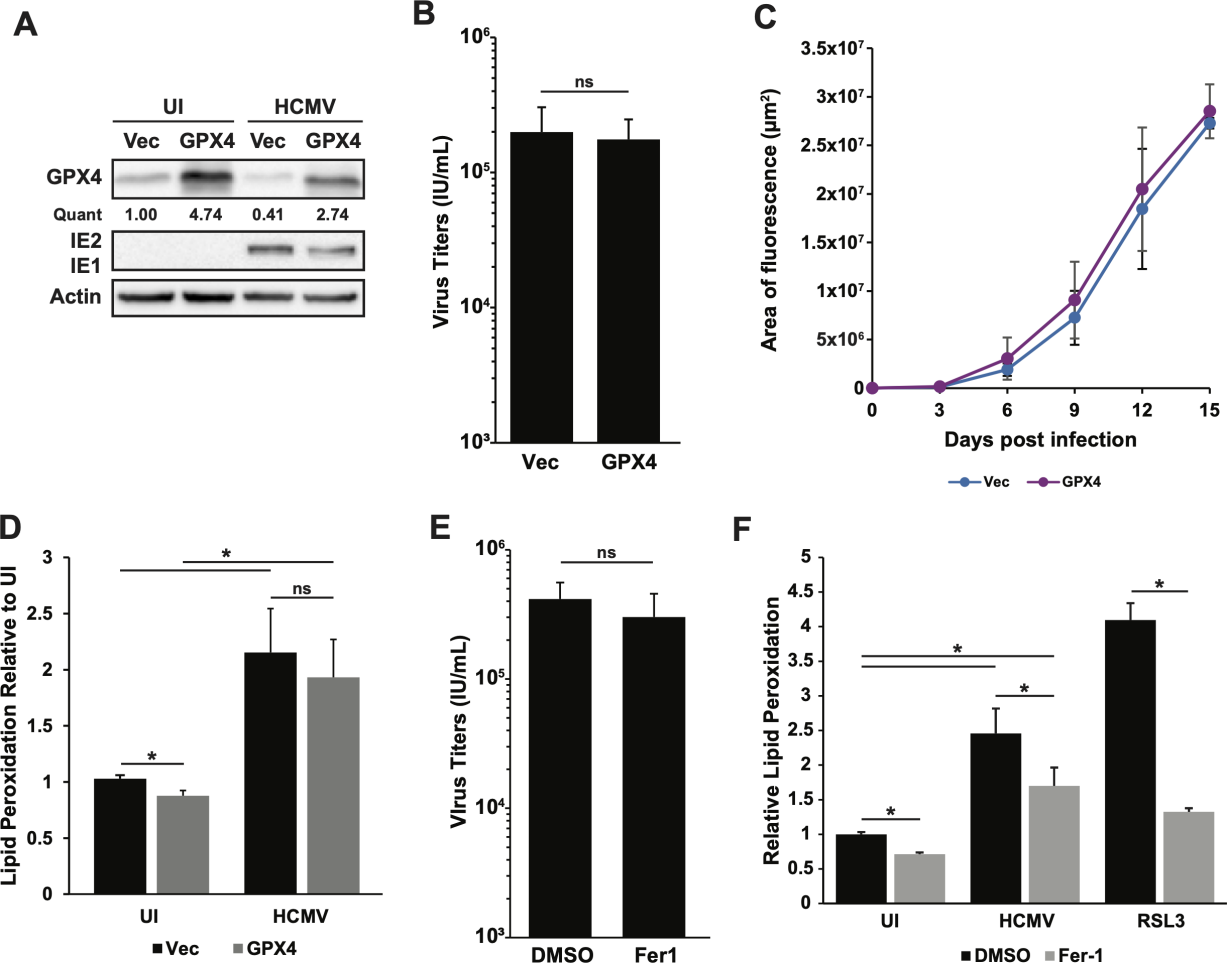
HCMV-induced lipid peroxidation is sensitive to ferrostatin-1 but not GPX4. (**A**) Western blot analysis of lysates from MRC-5 cells expressing an empty vector (Vec) or GPX4 and either uninfected or infected with a GFP-tagged TB40/E-WT virus (MOI = 3) and harvested at 96 hpi. Blots were probed for GPX4, IE1, IE2, and actin as a loading control. The values under the GPX4 blot denote quantification of each GPX4 band in the blot, relative to the GPX4 band in the uninfected vector cells. (**B**) Infectious titers at 120 hpi of MRC-5 cells expressing an empty vector or GPX4 and infected with TB40/E-WT at MOI = 3. Results are from two independent experiments, *n* = 4. (**C**) Analysis of HCMV spread in MRC-5 cells expressing an empty vector or GPX4 and infected at a low MOI (0.05) with a GFP-tagged TB40/E-WT virus (TB40/E-IE2AGFP). The fluorescence area (μm^2^) was measured every 3 days post-infection to monitor spread. Results are from two independent experiments, *n* = 5. (**D**) Lipid peroxidation measured by C11 BODIPY FITC fluorescence shift in MRC-5 cells expressing either an empty vector or GPX4. Lipid peroxidation was measured in both uninfected cells and cells infected as described in panel **B** at 96 hpi. Results are from two independent experiments, *n* = 6. (**E**) Infectious titers at 120 hpi of MRC-5 cells infected as described in panel **B** and treated with either DMSO or 1 µM ferrostatin-1 (Fer1) at both 3 and 72 hpi. Results are from two independent experiments, *n* = 3. (**F**) Lipid peroxidation measured at 72hpi by C11 BODIPY FITC fluorescence shift in MRC-5 cells either uninfected or infected as described in panel **B** that were treated for 48 hours with either DMSO or 1 µM ferrostatin-1 (Fer-1). As a positive control, uninfected MRC-5 cells were treated with 1 µM RSL3 with either DMSO or 1 µM ferrostatin-1 (Fer-1) for 4 hours before collection. Results are from two independent experiments, *n* = 6. For the above panels, * indicates *P* < 0.05 and ns = not significant.

The increase in lipid peroxidation observed during HCMV infection correlates with a decrease in GPX4 expression, which suggests a role for the downregulation of GPX4 in the increase of lipid peroxidation. To explore the relationship between GPX4 and the increased lipid peroxidation in infected cells, we stably transduced MRC-5 cells with an empty vector or GPX4-expressing lentivirus, which overexpressed GPX4 in both uninfected and infected cells (Fig. 6A). We measured viral titers, virus spread following low MOI infection, and levels of lipid peroxidation in the cells expressing the empty vector or GPX4-expressing vector. GPX4 overexpression did not affect virus production in a high MOI infection (Fig. 6B). We assayed virus spread following a low MOI infection using a recombinant virus encoding a green fluorescent protein (GFP) in frame following the viral immediate-early protein 2 via a T2A peptide (TB40/E-IE2AGFP). GFP signal was measured as an accurate surrogate for the spread of the virus through the monolayer (27, 40). As with high MOI infection, we found no effect of GPX4 overexpression upon virus spread following a low MOI infection (Fig. 6C). Interestingly, while GPX4 overexpression was accompanied by a slight decrease in basal levels of lipid peroxidation in uninfected cells, there was no significant reduction in lipid peroxidation in GPX4-expressing cells during infection (Fig. 6D). These results suggest that downregulation of GPX4 is required neither for productive infection nor for increased generation of lipid peroxides during HCMV infection.

As GPX4 overexpression did not reduce lipid peroxidation during HCMV infection, we sought to investigate whether other mechanisms that regulate levels of lipid peroxides impacted HCMV replication or induction of ferroptotic markers. To do this, we used the ferroptosis-inhibiting compound ferrostatin-1. Ferrostatin-1 is a synthetic antioxidant that directly reduces lipid peroxides by scavenging alkoxyl radicals and is regenerated by ferrous iron (41, 42). Ferrostatin-1 treatment had no impact on viral titers (Fig. 6E). However, in contrast to GPX4 overexpression, ferrostatin-1 treatment was able to significantly reduce lipid peroxidation levels in HCMV-infected cells, although they were not decreased to levels seen in uninfected cells (Fig. 6F). Ferrostatin-1 is known to inhibit lipid peroxidation induced by RSL3 treatment, so RSL3 was used as a positive control (Fig. 6F).

**Fig 7:**
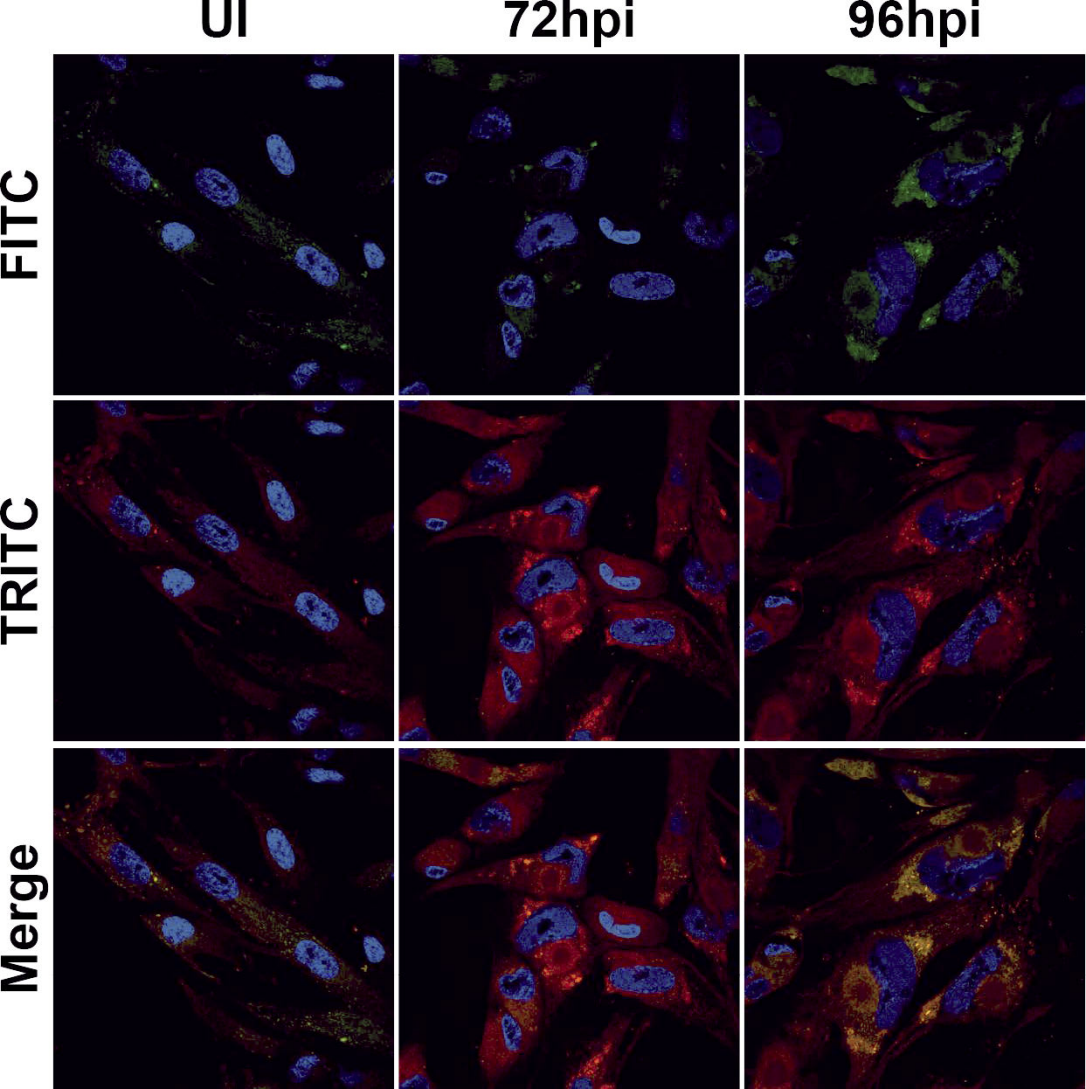
Oxidized lipids are not associated with the cVAC. Fluorescence images from confocal microscopy analysis of uninfected or HCMV-infected MRC-5 cells (TB40/E-WT at MOI = 3) harvested at 72 or 96 hpi. Cells were stained with 5 µM C11 BODIPY (581/591), then fixed, and nuclei were stained with DAPI (blue). The red channel shows total lipids, and the green channel shows lipid peroxides.

These data suggest that GPX4 does not regulate the lipid peroxidation that occurs during HCMV infection. However, the reduced expression of GPX4 and the increase in lipid peroxides made within infected cells could still potentially be used as a mechanism to either target virion membranes to reduce their fitness or to target HCMV-infected cells in patients by conferring susceptibility to the induction of ferroptosis within these cells. As lipid peroxides decrease membrane flexibility, it is possible that their incorporation into virion membranes could produce less fit virions. To explore this possibility, we examined whether lipid peroxides were concentrated within the cytoplasmic viral assembly compartment (cVAC), which is the site of virion assembly and envelopment. To do this, we visualized lipids and lipid peroxides using the C11 BODIPY (581/591) dye described above (Fig. 3B), which allowed us to see total lipids in red and lipid peroxides in green using confocal microscopy. There was a marked increase in green fluorescence, signifying lipid peroxides, at 72 and 96 hpi (Fig. 7), as quantified above (Fig. 3B). However, although there was clearly some green lipid peroxide staining in the circular cytosolic ring structures that readily identify the cVAC, the strongest green lipid peroxide staining at 96 hpi was in areas distinct from the cVAC (Fig. 7). Therefore, it is unlikely that lipid peroxidation is a process that is closely associated with virion assembly within infected cells.

**Fig 8:**
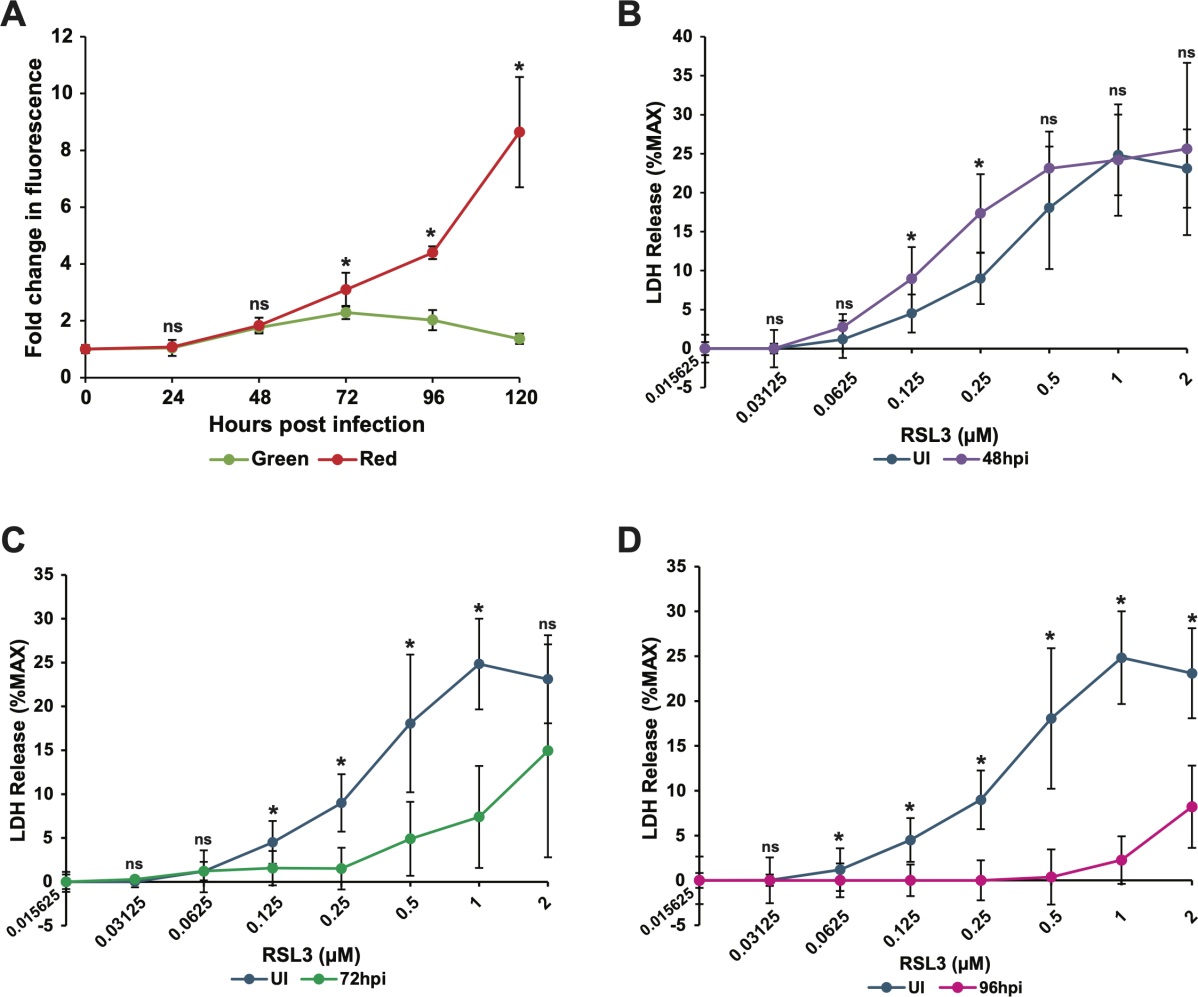
Ferroptosis is differentially regulated during HCMV infection. (**A**) Lipid peroxidation and total lipid measured with C11 BODIPY FITC (green) and phycoerythrin (PE) (red) fluorescence in MRC-5 cells either uninfected (0 hpi) or TB40/E-WT (MOI = 3) infected at 24, 48, 72, 96, and 120 hpi. Fluorescence is shown relative to uninfected cells. Results are from two individual experiments, *n* = 6. (**B**) LDH release measured in uninfected MRC-5 cells or infected MRC-5 cells (as described in panel A) at 48 hpi following 24 hour treatment with RSL3 at twofold serial dilutions from 2–0.015625 µM. Data are shown as the percentage of LDH release relative to the maximum LDH levels with the equation (experimental LDH – spontaneous LDH)/(maximum LDH – spontaneous LDH). Results are from two independent experiments, *n* = 6. (**C**) LDH release measured in uninfected MRC-5 cells infected MRC-5 cells (as described in panel A) at 72 hpi following 24-hour treatment with RSL3 at twofold serial dilutions from 2–0.015625 µM. Data are shown as described in panel **B**. Results are from two independent experiments, *n* = 6. (**D**) LDH release measured in uninfected MRC-5 cells or infected MRC-5 cells (as described in panel A) at 96 hpi following 24-hour treatment with RSL3 at twofold serial dilutions from 2–0.015625 µM. Data are shown as described in panel **B**. Results are from four independent experiments, *n* = 12. For the above panels, * indicates *P* < 0.05 and ns = not significant.

Notably, there was also a marked increase in red fluorescence, denoting total lipids, at 72 and 96 hpi (Fig. 7). The ability of lipid peroxides to mediate toxic effects in the cell is at least partially dependent on the reduced membrane flexibility that is a result of their build-up in cellular membranes. The ratio of lipid peroxides to total lipids is the controlling factor in mediating changes in membrane flexibility. Thus, if level lipid peroxides increases but simultaneously remains at the same ratio relative to total lipids, there will be no alteration in membrane flexibility. Therefore, we used the C11 BODIPY fluorescence assay to examine both bulk levels of lipids (in red) and levels of lipid peroxides (in green) over a time course of infection (36). We found that levels of bulk (red) lipids increased in HCMV-infected cells starting at 48 hpi and continued to increase through 120 hpi. (Fig. 8A). These data are supported by previous findings of increased fatty acid biogenesis during HCMV infection (12, 13). Lipid peroxides in HCMV-infected cells did increase at 48 and 72 hpi, decreasing thereafter (Fig. 8A). However, the ratio of lipid peroxides (green) to total lipids (red) never exceeded that found in uninfected cells, and by 96 and 120 hpi, the ratio of lipid peroxides to total lipids was much lower than in uninfected cells (Fig. 8A).

We next examined whether the increased levels of total lipids relative to lipid peroxides changed the ability of GPX4 inhibition to trigger ferroptosis-induced cell death by treating with the GPX4 inhibitor RSL3 and measuring cytotoxicity using the LDH release assay. We treated uninfected or HCMV-infected cells at 48, 72, or 96 hpi with titrated doses of RSL3 and assayed cell viability/cytotoxicity. At 48 hpi, a time point at which the lipid peroxides/lipid ratio was similar to that found in uninfected cells (Fig. 8A), HCMV-infected cells were marginally more sensitive to the induction of cell death with RSL3 than uninfected cells at doses within the linear range of RSL3 activity in this assay (Fig. 8B). However, at both 72 and 96 hpi, when there are substantially more total lipids relative to lipid peroxides (Fig. 7 and 8A), HCMV-infected cells were substantially less sensitive to induction of cell death with RSL3 (Fig. 8C and D). Indeed, at 96 hpi, uninfected cells were ~10-fold (26%–2.6%) more sensitive to 1 µM RSL3 treatment than infected cells at 96 hpi (Fig. 8D). As GPX4 expression is reduced to about one-third of normal expression at 96 hpi, this implies that infected cells are actually ~30-fold more resistant to RSL3 than uninfected fibroblasts at this time point, despite displaying markedly higher levels of lipid peroxides (Fig. 8D). Therefore, the correlation with increased lipid content may explain the resistance of infected cells to RSL3. Thus, while lipid peroxidation is stimulated during HCMV infection, infected cells may modulate bulk lipid biogenesis, rendering infected cells resistant to at least some mechanisms responsible for the induction of ferroptosis.

## DISCUSSION

HCMV dysregulates or counteracts many cellular pathways to facilitate infection. Here, we show that HCMV significantly increased lipid peroxidation above levels found in uninfected cells. This observation correlated with HCMV downregulation of GPX4, an enzyme that controls lipid peroxidation. Several viruses have been shown to induce lipid peroxidation and ferroptosis during infection. Newcastle disease virus, H1N1 influenza A virus, and H1N1 swine influenza virus are all associated with increased lipid peroxidation and decreased GPX4 expression (43–45). Herpes simplex virus 1 induces ferroptosis in astrocytes and microglial cells, including causing characteristic increases in iron, lipid peroxidation, and cell death (46). While we have previously demonstrated increased lipid peroxidation in HCMV-infected cells during late infection, here we expanded our understanding to establish that lipid peroxidation is stimulated by 48 hpi and significantly increased above uninfected levels through 120 hpi. To decipher the role for and mechanism of this increase in lipid peroxidation, we examined two well-known regulators of ferroptosis: GPX4 and FSP1. GPX4 is a phospholipid hydroperoxidase that uses GSH substrate to reduce lipid peroxides to lipid alcohol; FSP1 is an oxidoreductase that reduces coenzyme Q10 and halts the accumulation of lipid peroxides in cells lacking GPX4. We found no role for FSP1 in the modulation of lipid peroxidation in HCMV-infected cells, implying that GPX4 would be expected to bear the brunt of controlling lipid peroxidation within infected cells. However, although further inhibition of GPX4 with a compound (RSL3) or shRNA knockdown impaired virus infection, overexpression of GPX4 affected neither infection nor lipid peroxidation. Therefore, the evolutionary advantage that HCMV gains from downregulation of GPX4 remains unclear. HCMV-mediated GPX4 downregulation could be a side effect of other processes that are advantageous for virus replication or propagation. Alternatively, GPX4 downregulation may be required for efficient HCMV replication in cell types other than the fibroblasts examined here, such as monocytes. Extensive further studies would be required to investigate this possibility in multiple representative cell types. Nonetheless, these results indicate that the HCMV induction of lipid peroxidation proceeds via a novel and hitherto undescribed cellular or viral pathway.

The unexpected GPX4-independent increase in lipid peroxidation led us to examine both the regulatory pathways that affect both GPX4 function and other modulatory pathways that control lipid peroxidation. GPX4 requires GSH to reduce lipid peroxides. We observed that inhibition of GPX4 with RSL3 inhibited virus replication, while restriction of GSH supply via treatment with erastin did not. Erastin inhibits cystine import into the cell by inhibiting the action of system Xc^−^, thus depleting cellular GSH. This suggests that HCMV may modulate the pathway between system Xc^−^, the target of erastin, and GSH usage by GPX4. GSH is the most abundant low molecular weight, non-protein thiol, which are cysteine-containing compounds that regulate cellular redox homeostasis (47, 48). Previous work found that intracellular-free thiols concentrations increased during HCMV infection (39). As GSH is the most abundant free thiol in the cell, this suggested that GSH levels increased during HCMV infection. However, we measured GSH directly and found that levels decreased during HCMV infection. This implies that there are increased levels of alternative non-protein thiols during infection. While erastin ablated GSH levels in uninfected fibroblasts, GSH levels were reduced but still detectable in infected cells following erastin treatment. Therefore, an alternative pathway, such as *de novo* cysteine synthesis through the transsulfuration pathway, likely produces cysteine for GSH production during HCMV infection. The transsulfuration pathway enzymatically converts methionine into cysteine, which can then be used to synthesize GSH (49). This pathway is typically not very active outside of hepatocytes but could be induced in other cell types by HCMV infection (50, 51). *De novo* cysteine synthesis may significantly contribute to glutathione levels during infection, compensating for the loss of system Xc^−^ function during erastin treatment and maintaining glutathione levels to prevent cell death by ferroptosis (52, 53).

As the HCMV-induced increase in lipid peroxidation occurred independently of GPX4, we examined whether it could be inhibited by other modulators, and whether this process was, as we originally surmised, iron dependent. Ferrostatin-1 is a synthetic antioxidant that reduces lipid peroxides produced by RSL3 or erastin treatment but not by other treatments such as hydrogen peroxide or staurospaurine treatment (41, 42). Further intricacy in the lipid peroxidation pathway during HCMV infection was revealed when ferrostatin-1 treatment, but not GPX4 overexpression, reversed HCMV-induced lipid peroxidation. Ferrostatin-1 activity is regenerated by ferrous iron, and iron metabolism is intricately linked to the induction of lipid peroxidation and, downstream, to the induction of ferroptosis. Iron levels are increased in HCMV-infected cells at 48 hpi, when lipid peroxidation first increased, and decrease to normal levels thereafter (14). Notably, this increase in iron levels occurs at a stage of infection when HCMV-infected cells are more susceptible to the induction of ferroptotic cell death. Therefore, it is possible that the increased iron levels are driving susceptibility to ferroptosis. However, treatment with iron chelators ablates HCMV infection, so it is not possible to directly examine the role of iron in the induction of ferroptosis in the context of HCMV infection.

Although HCMV-infected cells are marginally more susceptible to cell death induced by RSL3-mediated GPX4 inhibition at 48 hpi, in all of the assays we performed, infected cells became less susceptible to ferroptotic cell death as infection progressed. By 96 hpi, infected cells are ~10 times more resistant to RSL3 cytotoxicity, despite GPX4 expression at only one-third of its levels in uninfected cells. This may be a result of HCMV-modulated iron expression, as outlined above. However, there are also other potential explanations. First, HCMV may deploy novel mechanisms that specifically counteract ferroptotic cell death. The observation that key regulators of ferroptosis do not regulate lipid peroxidation to their normal extent in HCMV-infected cells supports this possibility. A second, and more likely, possibility is that the increase in lipid biogenesis during HCMV infection (12, 13, 54) dilutes the effects of lipid peroxides and thus prevents the induction of ferroptosis. Increased lipid synthesis and elongation are required to supply the lipids required to form new virions (12, 54, 55). Our data demonstrate that the ratio of lipid-to-lipid peroxides in infected cells remains similar to that in uninfected cells until 48 hpi, at which point the proportion of oxidized lipids declines for the remainder of the infection examined. It is theorized that there is a threshold of lipid peroxidation that cells can tolerate before inducing cell death, but the limit is unknown (56). Consequently, despite the increase in lipid peroxidation in infected cells, it is possible that HCMV-infected cells do not undergo ferroptotic cell death because the threshold for tolerance of lipid peroxidation is increased. Alternatively, changes to the content of the lipidome during infection could also impact the sensitivity of infected cells to the induction of ferroptosis. However, drawing a conclusion about the definitive causation of this change in sensitivity to ferroptosis during infection would require further study. Our findings are the first example of the modulation of ferroptosis during HCMV infection. However, numerous previous studies have demonstrated examples of manipulation of cell stress responses during HCMV infection. For example, HCMV induces oxidative stress but also induces the activity of the transcription factors nuclear factor erythroid 2 related factor 1 (NRF1) and nuclear factor erythroid 2 related factor 2 (NRF2) to increase cellular resistance to oxidative stress during infection (1, 2). HCMV also induces endoplasmic reticulum stress that activates unfolded protein response; however, by inducing this stress response, the virus also activates protein kinase R (PKR)-like endoplasmic reticulum kinase which in turn stimulates lipogenesis that will benefit virus replication (3–5). In addition to modulating stress responses, HCMV also actively subverts cell death pathways, such as apoptosis. HCMV encodes two viral proteins, UL37 × 1 and UL36, which independently inhibit apoptosis during infection (6, 7). Thus, our findings that HCMV induces lipid peroxidation yet resists cell death via ferroptosis add to the growing list of cellular stress pathways that are modulated during HCMV infection in a way that supports, rather than hinders, replication.

Together, these findings demonstrate that the study of lipid peroxidation and ferroptosis during HCMV, and potentially other virus infections, must be carefully interpreted. The lack of direct measures of ferroptosis means that the interpretation of correlates of ferroptosis could result in misleading conclusions. For instance, during HCMV infection, a large decrease in GPX4 expression, aligned with a corresponding increase in lipid peroxidation, could readily lead to the conclusion that HCMV-infected cells are more susceptible to induction of ferroptosis. However, we found the opposite to be true. Induction of ferroptosis in HCMV-infected cells involves novel virus-modulated pathways, including GPX4-independent control of lipid peroxidation and non-classical synthesis of the GPX4 substrate, GSH.

## MATERIALS AND METHODS

### Cell culture, virus stocks, and infections

Normal human dermal fibroblasts (HDFs; Cell Applications Inc.) and MRC-5 cells (ATCC) were cultured as previously described (27). All cytomegalovirus stocks were generated from bacterial artificial chromosomes (BACs) (57), and virus stocks were propagated by electroporating purified BAC DNA into MRC-5 cells according to published protocols (58) and titrated by the immunological detection of immediate-early proteins as previously described (59, 60). Wild-type TB40/E or the IE-2A-GFP recombinant virus was used for all experiments (40, 61).

For HCMV infections, virus was added to HDFs or MRC-5 cells and allowed to incubate for 3 hours at 37°C. Where applicable, virus was UV inactivated under a Mineralight ultraviolet lamp (shortwave UV at 254  nm) for 30 min at a distance of 10 cm. Cells were washed twice with 1× phosphate buffered saline (PBS) and replaced with fresh growth medium. Single-step growth curve analysis of all HCMV viruses was performed on MRC-5 cells infected at MOI = 3. Virus was harvested by scraping cells in the medium, sonicating, vortexing, and centrifuging at 13,000 rpm for 10 min at 4°C. Supernatants were collected, aliquoted, flash-frozen in liquid nitrogen, and stored at −80°C until analysis.

Lentivirus was generated using the third-generation packaging system: pCMV-VSV-G (gift from Bob Weinberg, Addgene #8454), pMDLg-RRE (gift from Didier Trono, Addgene #12251), and pRSV-Rev (gift from Didier Trono, Addgene #12253) as described previously (62). The FSP1 (TRCN0000064424 [clone #1], TRCN0000064426 [clone #2], and TRCN0000064426 [clone #3]) and GPX4 (TRCN0000046250 [clone #1] and TRCN0000046252 [clone #2]) shRNA plasmids were from the TRC1.0 shRNA library. Scrambled and GFP controls were a gift from David Sabatini (Addgene #1864 and #30323). Primers and gene fragments used for all cloning are listed in Table 1. The GPX4 3′UTR sequence was synthesized (Integrated DNA Technologies) including XhoI/NotI sites and inserted into pCDH-CMV-MCS-EF1-Puro (System Biosciences) to make pCDH-3′GPX4. The GPX4 sequence was amplified from fibroblast cDNA and inserted into the NheI/EcoRI sites on pCDH-3′GPX4 to generate pCDH-GPX4. Luciferase plasmids were made from pTRE3G-Bi-Luc (Takaro Biosciences). The CMV promoter (major immediate early promoter) was amplified from TB40/E BAC DNA with primers to add KpnI/XhoI sites and inserted into pTRE3G-Bi-Luc to make pTRE3G-MIEP-Luc. The MIEP-Luc sequence was then amplified with primers including SpeI/XhoI sites and inserted into pCDH-IRES-EGFP to make pCDH-MIEP-Luc-IRES-EGFP. The synthesized GPX4 3′UTR was inserted into pCDH-MIEP-Luc-IRES-EGFP using XhoI/NotI to make pCDH-MIEP-Luc-3′GPX4. As a control, the SV40 terminator sequence was amplified from pEGFPC1 with primers containing XbaI/NotI and inserted into pCDH-MIEP-Luc-IRES-EGFP to make pCDH-MIEP-Luc-SV40t.

**TABLE 1:** Nucleotide sequences of primers used for PCR

Name	Sequence
NheI-GPX4-For	GCATGCTAGCATGAGCCTCGGCCGCCTT
EcoRI-GPX4-Rev	GCATGAATTCCTAGAAATAGTGGGGCAG
XbaI-EGFP-For	GCATTCTAGAATGGTGAGCAAGGGCGAG
NotI-SV40t-Rev	GCATGCGGCCGCTAAGATACATTGATGAGT
KpnI-MIEP-For	GCATGGTACCCTGGAATACGACAAGATAAC
XhoI-MIEP-Rev	GCATCTCGAGGGATCGGTCCCGGTGTCT
NheI-MIEP-For	GCATGCTAGCCTGGAATACGACAAGATAAC
XhoI-Luc-Rev	GCATCTCGAGTTACAATTTGGACTTTCC
3′GPX4	CTCGAGCTCCACAAGTGTGTGGCCCCGCCCGAGCCCCTGCCCACGCCCTTGGAGCCTTCCACCGGCACTCATGACGGCCTGCCTGCAAACCTGCTGGTGGGGCAGACCCGAAAATCCAGCGTGCACCCCGCCGGAGGAAGGTCCCATGGCCTGCTGGGCTTGGCTCGGCGCCCCCACCCCTGGCTACCTTGTGGGAATAAACAGACAAATTAGCCTGCTGGATAAAAGCGGCC
GPX4-For	ACCGAAGTAAACTACACTCAG
GPX4-Rev	GGCGAACTCTTTGATCTCTT
GAPDH-For	ACCCACTCCTCCACCTTTGAC
GAPDH-Rev	CTGTTGCTGTAGCCAAATTCGT

For lentivirus transduction, sub-confluent fibroblasts were seeded overnight and transduced with lentivirus and 8 µg/mL polybrene (Sigma Aldrich). Transduced cells were selected using 2 µg/mL puromycin (Thermo Fisher) before seeding for HCMV infection as described above.

### Western blotting

Western blots and cell lysate harvests were performed as previously described (62). Where applicable, 100 µg/mL acyclovir (EMD Millipore) was added at 3 hpi. The primary antibodies are listed in Table 2. The horseradish peroxidase-conjugated secondary antibodies were from GE Healthcare and Fisher Scientific. Blots were developed with SuperSignal West Pico PLUS Chemiluminescent Substrate (Thermo Fisher Scientific).

**TABLE 2:** Sources of primary antibodies used for western blot analyses

Target	Source
IE1/2	(59)
IE1/2 (ex2/3)	Jim Alwine (63)
FSP1	Proteintech
pp28	Virusys
GPX4	CellSignaling
Actin	MilliporeSigma
p115	Proteintech

### Reverse transcriptase and quantitative PCR

At indicated time points, RNA was isolated from cells using RNeasy Mini kit (Qiagen) following the manufacturer’s protocol. RNA concentration was quantified using NanoDrop 2000 (ThermoFisher), and cDNA was synthesized using SuperScript First-Strand Synthesis System for RT-PCR (ThermoFisher) following the manufacturer’s protocol. Samples were cycled on a StepOnePlus Real-Time PCR System (ThermoFisher): 50°C 2 min, 95°C 10 min, (95°C 15 s, 60°C 60 s, 55°C 30 s, and 40 cycles). Primers are listed in Table 1. Data were analyzed using the delta-Ct method with GAPDH as a normalization control.

### Cell Titer Glo and LDH cytotoxicity assays

ATP levels were evaluated with the Cell Titer-Glo cell viability assay (Promega) following the manufacturer’s instructions. Cytotoxicity and membrane integrity were analyzed using the CyQUANT assay following the manufacturer’s instructions (Thermo Fisher Scientific).

### C11 BODIPY lipid peroxidation assay

Lipid peroxidation was measured using the lipophilic oxidizable fluorescent molecule C11 BODIPY (581/591; Thermo Fisher Scientific) which shifts fluorescence excitation/emission from 581/591 to 488/510 upon lipid peroxidation. Following infection or treatment, MRC-5 cells were stained with 5 µM C11 BODIPY in 10% Dulbecco's modified Eagle medium (DMEM) for 30 min at 37°C. Cells were then trypsinized and collected by centrifugation at 200 × *g* for 4 min at 4°C, followed by washing with 0.2% bovine serum albumin (BSA) in 1× PBS and fixation with 4% paraformaldehyde on ice for 15 min. Cells were again collected by centrifugation and washed with 5% glycine in 1× PBS, followed by 0.2% BSA. C11 BODIPY fluorescence shift was measured via flow cytometry on LSR Fortessa (BD Biosciences) in the Penn State College of Medicine Flow Cytometry Core. Data analysis was performed with FlowJo software (BD Life Sciences), and the geometric mean fluorescence intensity for FITC was calculated for live cells.

### Luciferase assay

Luciferase activity was measured using the Promega luciferase assay (Promega). Briefly, cells were transduced and selected as described with luciferase-expressing lentiviruses containing a control SV40 terminator sequence or the GPX4 3′UTR sequence. The following selection, cells were infected or mock-infected and harvested 24 hpi using 5× lysis buffer following the manufacturer’s protocol. To measure luciferase activity, the substrate was mixed with the sample, and luminescence was quantified with a luminometer (Berthold Detection Systems). Luminescence is shown relative to SV40 terminator luciferase activity.

### Cell treatments (RSL3, erastin, ferrostatin-1, deferoxamine, etc.)

All compounds were resuspended in DMSO and then diluted in media to the final indicated concentrations. DMSO was used as a vehicle control. RSL3 (Cayman Chemicals) was used at a concentration of 1 µM except where indicated and added 24 hours before harvest on infected cells and 24 or 4 hours before harvest on uninfected cells. Erastin (Cayman Chemicals) was used at a concentration of 5 µM except where indicated and added 24 hours before harvest on infected cells or 14 hours before harvest on uninfected cells. Ferrostatin-1 (Cayman Chemicals) was used at a concentration of 1 µM and added to infected cells at the time of infection and 72 hpi for viral titer measurements and at 24 hpi or 4 hours before harvest, where indicated for lipid peroxidation measurements. Deferoxamine mesylate (Cayman Chemicals) was used at a concentration of 100 µM and added to cells at 24 hpi or 4 hours before harvest where indicated. Tri-1 (MedChemExpress) was used at a concentration of 1 µM and added to infected cells at 72 hpi.

### Spread assays

Cells were infected at MOI = 0.05 with the TB40/E-IE-2A-GFP virus (40). Infection is imaged every 3 days with confocal microscopy on a Nikon Eclipse Ti inverted microscope until the monolayer is totally infected. The fluorescence area is quantified on NIS Elements (Nikon Instruments Inc.) throughout infection.

### Confocal microscopy

Fibroblasts were grown on coverslips and infected with HCMV as described above. Cells were stained with 5 µM C11 BODIPY (581/591; ThermoFisher Scientific) in 10% DMEM for 30 min at 37°C. Cells were then fixed with 2% paraformaldehyde for 15 min at room temperature. Slides were then stained with DAPI (0.1 µg/mL) for 10 min at room temperature and mounted with ProLong Diamond Antifade Mountant (ThermoFisher). Images were taken on a C2+ Confocal Microscope System (Nikon). Images were processed using NIS elements software and are shown as a single slice of a *Z*-stack.

### TXNRD activity assay

TXNRD activity was evaluated with a thioredoxin reductase assay kit (Abcam) following the manufacturer’s instructions.

### Statistical analyses

Statistical analysis was performed using Student’s *t*-test in Microsoft Excel and GraphPad Prism.

## Data Availability

This paper does not contain new nucleotide sequences, high throughput data, or structural information. All data associated with the article are freely available via the Penn State repository at ScholarSphere under https://doi.org/10.26207/ehd2-v087.

## SUPPLEMENTAL MATERIAL

Figure S1 jvi.01851-24-s0001.docx RSL3 off-target effect on TXNRD does not affect viral replication.

ASM does not own the copyrights to Supplemental Material that may be linked to, or accessed through, an article. The authors have granted ASM a non-exclusive, world-wide license to publish the Supplemental Material files. Please contact the corresponding author directly for reuse.
