# Supplementary material for: HCMV infection downregulates GPX4 and stimulates lipid peroxidation but does not induce ferroptosis
Source: J Virol. 2025 Jan 7;99(2):e01851-24. doi: 10.1128/jvi.01851-24 (PMC11852782; doi:10.1128/jvi.01851-24)
Supplement: Figure S1 — RSL3 off-target effect on TXNRD does not affect viral replication. [file jvi.01851-24-s0001.docx]

**Supplemental Figure**

**
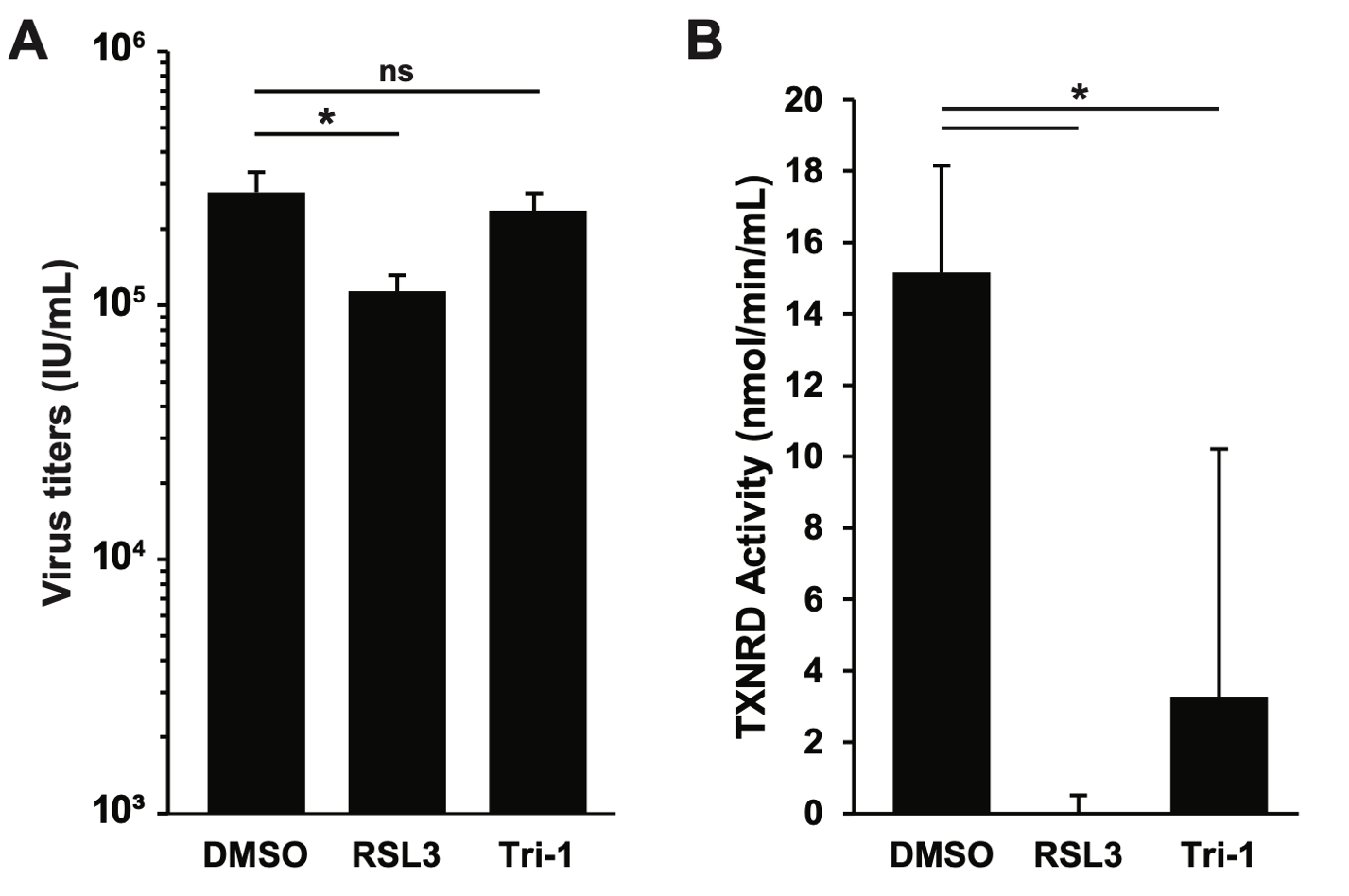
**

**Figure S1. RSL3 off-target effect on TXNRD does not affect viral replication.** (A) Infectious titers at 120 hpi from TB40/E-WT (MOI=3) infected MRC-5 cells treated with vehicle control (DMSO), RSL3 (1μM), or Tri-1 (1μM) at 72 hpi. Results are from 2 independent experiments, n=6. (B) TXNRD activity (in nmol/min/mL) in MRC-5 cells infected with TB40/E-WT (MOI=3) and treated with DMSO, RSL3 (1μM), or Tri-1 (1μM) at 72 hpi and harvested at 120hpi. Results are from 1 independent experiment, n=3. For the above panels, * indicates p<0.05, and ns = not significant.
